# Leadless Pacemaker vs. Transvenous Pacemaker in End Stage Kidney Disease: Insights from the Nationwide Readmission Database

**DOI:** 10.3390/jcm14010202

**Published:** 2025-01-02

**Authors:** Sajog Kansakar, Azka Naeem, Norbert Moskovits, Dhan Bahadur Shrestha, Jurgen Shtembari, Monodeep Biswas, Ghanshyam Shantha, Binaya Basyal, James Storey, Daniel Katz

**Affiliations:** 1 Department of Internal Medicine, Maimonides Medical Center, Brooklyn, NY 11219, USA; sajog.kansakar@gmail.com (S.K.); azkanaeem85@gmail.com (A.N.); 2 Department of Cardiology, Maimonides Medical Center, Brooklyn, NY 11219, USA; nmoskovits@maimo.org; 3 Division of Cardiology, Department of Internal Medicine, Bassett Medical Center, 1 Atwell Rd, Cooperstown, NY 13326, USA; james.storey@bassett.org (J.S.); daniel.katz@bassett.org (D.K.); 4 Division of Cardiology, Department of Internal Medicine, Carle Foundation Hospital, 611 W Park St, Urbana, IL 61801, USA; jshtembari@gmail.com; 5 Division of Cardiac Electrophysiology, Department of Internal Medicine, University of Maryland Medical Center, Baltimore, MD 21201, USA; mbiswasmd@gmail.com; 6 Division of Cardiac Electrophysiology, Department of Internal Medicine, East Carolina University, Greenville, NC 27858-4353, USA; ghanshyampalamaner@gmail.com; 7 Division of Cardiology, Medstar Heart and Vascular Institute, College Park, MD 200100, USA; binaya.basyal@medstar.net

**Keywords:** leadless pacemaker, end stage renal disease, Nationwide Readmission Database

## Abstract

**Background**: Leadless pacemakers offer a safe and effective alternative pacing strategy. However, limited data are available for patients with end stage renal disease (ESRD), a population of significant relevance. **Methods:** Using the Nationwide Readmission Database, we extracted data from all adult patients with ESRD who underwent traditional transvenous or leadless pacemaker implantation between 2016 and 2021. We compared in-hospital mortality, 30-day readmission rates, complication rates, and healthcare resource utilization between the two cohorts. **Results:** A total of 6384 (81.2%) patients were included in the transvenous pacemaker cohort, and 1481(18.8%) patients were included in the leadless pacemaker cohort. In patients with ESRD, leadless pacemaker implantation was linked to higher in-hospital complications when compared to transvenous pacemakers. These included the need for blood transfusion (aOR 1.85, 95% CI 1.32–2.60, *p* < 0.01), vascular complications (aOR 3.6, CI 1.40–9.26, *p* = 0.01), and cardiac complications (aOR 4.12, CI 1.70–9.98, *p* < 0.01). However, there were no differences between the two groups in terms of in-hospital mortality and 30-day readmission rates. The median length of stay was longer for leadless pacemaker implantation than transvenous pacemaker implantation (5 days vs. 4 days, *p* < 0.01). The total hospitalization charges were also higher ($139,826 vs. $93,919, *p* < 0.01). **Conclusions:** Although previous studies have demonstrated lower long-term complication rates with leadless pacemakers than transvenous pacemakers, our analysis shows a higher risk of short-term in-hospital complications in ESRD patients, though no differences in in-hospital mortality and 30-day readmissions.

## 1. Introduction

In 2020, the number of people in the United States living with end stage renal disease (ESRD) exceeded 800,000 with 69% of them on dialysis. This represents a substantial growth of about 35% over two decades [1]. Compared to the general population, bradyarrhythmia is more common in patients with ESRD, and they experience a higher rate of pacemaker implantation [2,3]. However, traditional transvenous pacemaker (TVPM) implantation in this population is complicated by venous access issues, bleeding risk, and high risk of device infection [4,5,6]. Thus, ESRD is linked to an elevated risk of complications of TVPM implantation (39% vs. 11%) as well as TVPM revision (29.5 vs. 18.6 per 1000-person years after implantation) [7,8].

Leadless pacemakers (LPM) do not have lead- or pocket-related complications, and, more importantly, can overcome infection and venous access issues, which is significant in ESRD [9,10]. Numerous observational studies have demonstrated a lower complication rate with LPM compared to TVPM; however, there is a lack of representation of ESRD patients in these studies [9,11,12,13,14,15]. Studies comparing TVPM and LPM in ESRD patients, a crucial demographic for LPM use, are limited [16,17]. To address this knowledge gap, we analyzed contemporary real-world data from the Nationwide Readmission Database (NRD) to investigate differences in in-hospital outcomes, healthcare resource utilization, and 30-day readmission rates in ESRD patients undergoing TVPM or LPM implantation.

## 2. Materials and Methods

We conducted a retrospective cohort study utilizing the Nationwide Readmission Database (NRD). The NRD was developed by the Agency for Healthcare Research and Quality (AHRQ) for the Healthcare Cost and Utilization Project (HCUP). The data can be procured from the HCUP website, and it is the most extensive publicly available all-payer database in the United States. The NRD incorporates discharge data from 30 US states, covering 61.2% of the US resident population and 59.6% of all US hospitalizations, encompassing about 16 million unweighted and 32 million weighted discharges in 2021. It utilizes deidentified linkage numbers to track each patient’s admission, discharge, readmission, and death within a year [18]. For our study, we utilized data from the NRD covering the years 2016 to 2021. Institutional Review Board approval was not necessary since the data were sourced from a deidentified administrative database.

**Figure 1:**
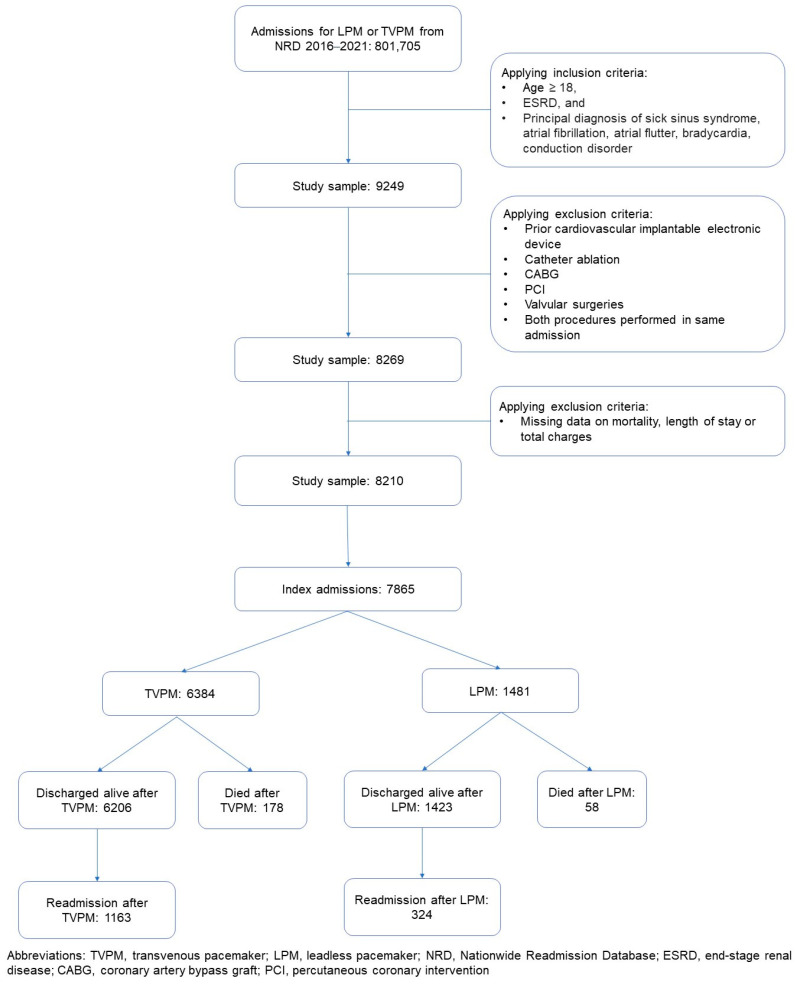
Patient selection flow diagram.

We identified our study population utilizing the diagnostic and procedural codes from the International Classification of Diseases—Tenth Revision (ICD-10). We sampled the NRD database to identify all admissions for adults (age ≥ 18 years) that either had implantation of LPM or TVPM (single or dual chamber), and had a principal diagnosis of sick sinus syndrome, atrial fibrillation, atrial flutter, bradycardia, or conduction disorder using ICD-10 codes that are summarized in Supplementary Table S1. We excluded admissions for patients with a history of a cardiovascular implantable electronic device (CIED) or if they had undergone procedures during the admission that could impact the outcomes of interest, e.g., coronary artery bypass grafting, catheter ablation, valve procedures, or percutaneous coronary intervention. Admissions with missing data on the primary outcome were excluded (<1%). Admissions were divided into two groups based on whether they underwent LPM or TVPM implantation on the index admission. The flow diagram for patient selection is summarized in Figure 1. We only included discharges that occurred between January and November to ensure a 30-day follow-up period post-discharge.

The primary outcomes were in-hospital mortality on the index admission and a 30-day readmission rate. The time to readmission was determined by counting the number of days from discharge to the readmission date. Only the first readmission within 30 days post-discharge was considered. Secondary outcomes were device complications, cardiac complications, vascular complications, blood transfusion, respiratory complications, acute kidney injury, length of stay, and total hospitalization charges (all during the index admission). We used ICD-10 codes to identify complications.

Data were analyzed after considering the stratification and discharge weights recommended by HCUP NRD guidelines to account for the complex survey design. Descriptive statistics were presented as frequency with percentage for categorical variables and median with interquartile range (IQR) for continuous variables. A T-test was utilized for continuous variables, while Pearson’s chi-square test was utilized for categorical variables. Binary outcomes were modeled using logistic regression, and continuous outcomes were modeled using negative binomial regression. We used multivariable analysis to adjust for all baseline characteristics at both the patient and hospital levels (sex, age, race, Charlson Comorbidity Index, median household income for patient’s ZIP code, insurance, region of hospital, hospital bed size, location/teaching status of the hospital, hypertension, diabetes, peripheral vascular disease, coronary artery disease, heart failure, chronic lung disease, chronic liver disease, obesity, smoking, malignancy, coagulopathy, and atrial fibrillation) to calculate the adjusted odds ratio (aOR). After adjusting for the same variables, we computed the hazard ratio (HR) to compare the association of TVPM or LPM with 30-day readmission in a multivariable Cox regression analysis. A two-tailed *p* value of less than 0.05 was deemed statistically significant. The analysis was conducted using STATA-MP, version 14.2 (StataCorp LLC, Lakeway Drive, TX, USA).

## 3. Results

After applying our inclusion and exclusion criteria, the study comprised a total of 7865 admissions, as summarized in the flow diagram in Figure 1. The study sample consisted of 3116 (39.6%) females with a median age of 72. The study had 6384 (81.2%) admissions for TVPM and 1481 (18.8%) admissions for LPM. The baseline characteristics for the two cohorts were similar; however, compared to TVPM admissions, LPM admissions were higher in hospitals with large bed sizes (71.0% vs. 59.4%, *p* < 0.01) and teaching hospitals (89.4% vs. 75.3% < 0.01), and had a higher burden of heart failure (58.5% vs. 51.6%, *p* < 0.01), atrial fibrillation (28.3% vs. 24.7%, *p* = 0.03), and coagulopathy (14.9% vs. 12.2%, *p* = 0.05). The comprehensive baseline characteristics can be found in Table 1.

In our study sample, there was no difference in mortality between TVPM and LPM admissions (2.8% vs. 3.7%, *p* = 0.31). However, LPM admission was associated with higher odds of cardiac complications (0.5% vs. 1.9%, *p* < 0.01), vascular complications (0.5% vs. 1.5%, *p* = 0.01), and need for blood transfusion (5.1% vs. 8.6%, *p* < 0.01). The two cohorts had no difference in device or respiratory complications and AKI. Compared to TVPM admissions, LPM admissions were associated with longer lengths of stay (5 days vs. 4 days, *p* < 0.01) and total hospitalization charges ($139,826 vs. $93,919, *p* < 0.01). The detailed in-hospital outcomes are given in Table 2.

**Figure 2:**
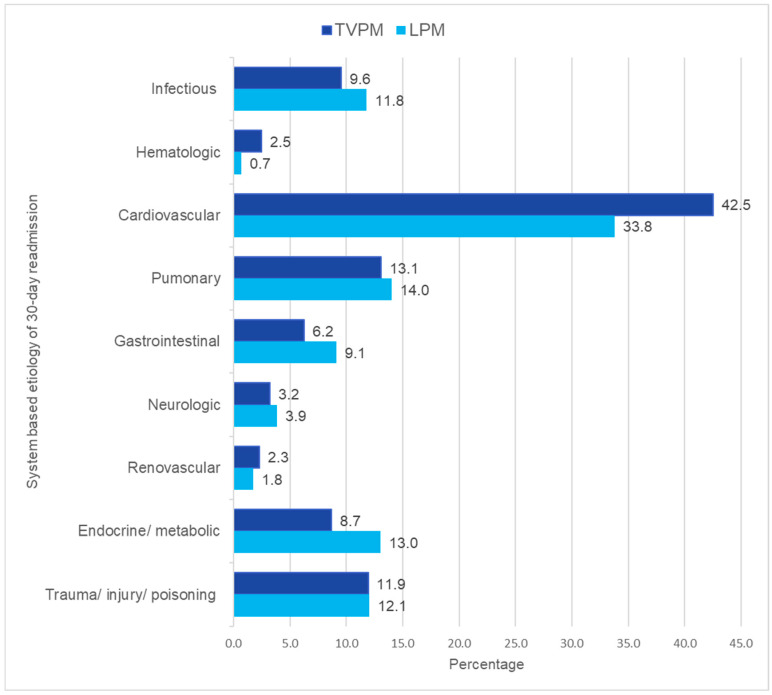
Breakdown of causes for 30-day readmissions by system.

Among TVPM admissions that were discharged alive, there were 1163 (18.7%) 30-day all-cause readmissions, while LPM admissions that were discharged alive had 324 (22.7%) 30-day all-cause readmissions, but no statistically significant difference was noted (adjusted HR 1.15, 95% CI 0.95–1.39, *p* = 0.15). Figure 2 compares the system-wise causes of 30-day readmissions for TVPM and LPM patients. Cardiovascular causes were the leading causes of readmission in both TVPM (42.5%) and LPM (33.8%). “Hypertensive heart disease and chronic kidney disease with heart failure” was the most common cardiac diagnosis for readmission in TVPM (20.7%) and LPM (25.4%).

**Figure 3:**
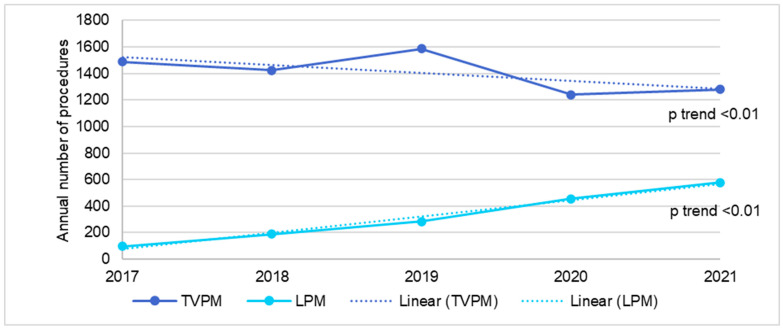
Trend in the annual number of transvenous or leadless pacemaker implantation in ESRD patients.

**Figure 4:**
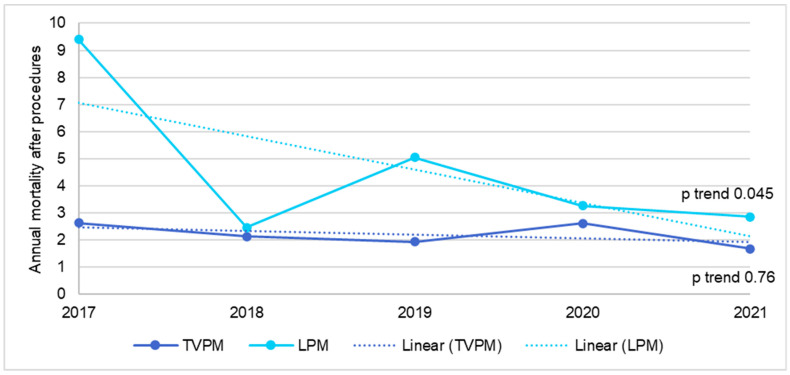
Trend in mortality for transvenous or leadless pacemaker implantation in ESRD patients.

**Figure 5:**
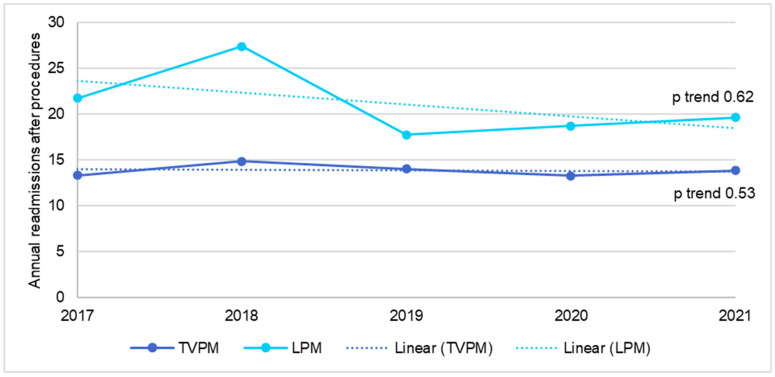
Trend in 30-day readmission for transvenous or leadless pacemaker implantation in ESRD patients.

In the ESRD population, the annual number of TVPM decreased from 1488 in 2017 to 1281 in 2021 (*p* trend < 0.01), while the annual number of LPM increased from 98 in 2017 to 580 in 2021 (*p* trend < 0.01), as shown in Figure 3. There was no change in mortality rate after TVPM during this time period (*p* trend= 0.76), while the mortality for LPM decreased from 9.4% in 2017 to 2.9% in 2021 (*p* trend= 0.045), as shown in Figure 4. There was no change in 30-day readmission rate for both TVPM (*p* trend= 0.62) and LPM (*p* trend= 0.53) during this time period (Figure 5).

## 4. Discussion

This is the first national-level study comparing in-hospital outcomes, healthcare utilization, and readmissions for TVPM and LPM in ESRD patients. The key findings of our study are as follows:

(1) In patients with ESRD undergoing either TVPM or LPM, there was no significant difference in in-hospital mortality rates (2.8% vs. 3.7%, *p* = 0.31).
(2) Compared to TVPM, LPM was associated with higher odds of cardiac complications (0.5% vs. 1.9%, aOR 4.11, 95% CI 1.69–10.3, *p* < 0.01), vascular complications (0.5% vs. 1.5%, 3.57, 95% CI 1.40–9.11, *p* < 0.01), and blood transfusion (5.1% vs. 8.6%, aOR 1.85, 95% CI 1.32–2.60, *p* < 0.01).
(3) Compared to TVPM, LPM was associated with a lengthier median hospital stay (4 days vs. 5 days) and total hospitalization charges ($93,919 vs. $139,826).
(4) The 30-day readmission rates did not differ between TVPM and LPM admissions. (18.7% vs. 22.7%, *p* = 0.14). Cardiovascular causes were the leading causes of readmission in both TVPM (42.5%) and LPM groups (33.8%).

Prior studies have demonstrated a significantly higher incidence of arrhythmias in ESRD patients due to the effect on potassium homeostasis, fluid, and electrolyte shifts with dialysis, increased left ventricular size, coronary artery disease, and autonomic imbalance [2]. Moreover, sudden cardiac death (SCD) is a major cause of mortality among ESRD patients, responsible for 25–30% of deaths [19,20]. Research employing implantable loop recorders has shown that bradyarrhythmia is responsible for up to 20% of SCDs in this population [10]. Thus, the role of cardiac pacing becomes crucial in this context. However, TVPM is associated with several lead and pocket-related complications, such as lead dislodgement, endocarditis, and pocket infection [6,21]. Vascular access and infections are especially important issues in ESRD. Having vascular leads can elevate the risk of venous thrombosis and stenosis, which can lead to a high rate of AV fistula failure [4,5,22]. Furthermore, the contralateral arm may not have a suitable vein available, which can limit options further. ESRD patients are also at increased risk of systemic infections with seeding in the pacemaker leads due to immunosuppression and frequent vascular access [23]. This can lead to device extraction, which has been associated with overall worse in-hospital and long-term outcomes including mortality in the general population [24,25] and in ESRD patients [26]. LPM can overcome these risks, which is crucial in the context of ESRD, as well as other lead- and pocket-related complications [21].

A previous study by Alhuarrat et al. that utilized National Inpatient Sample (NIS) data from 2016–2019 reported a higher in-hospital mortality for adults undergoing LPM (2.51%) compared to those undergoing TVPM (1.28%) (*p* < 0.01) [11]. However, in our study, which used NRD data from 2016–2021 and focused only on ESRD patients, there was no significant difference in mortality between the LPM and TVPM group. Our study also observed a trend in decreasing in-hospital mortality after LPM, from 9.4% in 2017 to 2.9% in 2021 (*p* = 0.045). This trend was also reported by Khan et al. [27]. The inclusion of more recent data, which shows a lower mortality rate for LPM, as well as a higher in-hospital mortality rate for TVPM in ESRD patients, might explain the discrepancy in in-hospital mortality between our study and that by Alhuarrat et al. Of note, both NIS and NRD databases are retrospective cohorts with a high risk of selection bias, likely with predominantly those patients getting LPM having significantly higher comorbidity/risk factors. Despite this substantially higher risk, the major adverse events, particularly in-hospital mortality and readmission rates, did not vary between the LPM and TVPM groups. The mortality rates were trending down with time. This signifies a significant improvement in LPM technology and outcomes even in such a complex patient population group, and a viable option where TVPM is either contraindicated or not feasible to implant safely.

Compared to TVPM, we also found higher odds of cardiac complications, vascular complications, and blood transfusion with LPM in ESRD patients. Studies have consistently demonstrated an elevated risk of pericardial effusion and cardiac perforation with LPM, which was demonstrated in our study as well [10]. The increased rates of vascular complications and blood transfusions observed in the LPM group in our study might be attributed to the requirement of using a large bore sheath through the femoral vein for LPM implantation. One prior study reported higher in-hospital complications such as vascular and cardiac complications, blood transfusion requirement, and venous thromboembolism with LPM compared to TVPM. In contrast, another study reported lower total in-hospital complication rates (8.6% with LPM vs. 11.2% with TVPM) [11,28]. These disparities in the result could be due to associated selection bias and heterogeneous patient populations in different studies.

These studies primarily address in-hospital mortality and complications. However, when it comes to long-term outcomes, the prevailing consensus in the current literature favors LPM. The pivotal Micra-CED study compared leadless and TVP over 3 years [29,30]. In this study, compared to TVPM (*n* = 10,212), patients with LPM (*n* = 6219) had 32% lower long-term complications, 41% lower rates of reintervention, and few infections and heart failure hospitalization rates. There was no difference in 3-year all-cause mortality. Similarly, a meta-analysis of observational studies calculated a pooled incidence of 1.77% with Micra at 1 year after implantation, with 51% lower odds of complications than with TVPM [31]. The post hoc analysis of the Micra-CED study compared the safety among LPM and TVPM in high-risk subgroups, which included ESRD patients too. It indicated that overall, ESRD patients experienced a higher frequency of both acute and chronic complications compared to other high-risk groups, but there were no disparities between leadless and transvenous pacemakers [17].

Our study found higher healthcare utilization for LPM admissions compared to TVPM admissions. The higher length of stay and hospitalization charges in LPM admissions could be related to the higher in-hospital complications observed in LPM admissions. Previous studies have similarly demonstrated higher healthcare utilization in LPM admissions [11]. We calculated the 30-day readmission rate in ESRD patients for TVPM (18.7%) and LPM (22.7%), which is higher than those calculated by studies not specifically targeting ESRD. One study calculated a 30-day readmission rate of 16% after LPM using NRD data from 2017–2019 [32]. ESRD patients are at a higher risk of complications from not only TVPM but also from LPM, which could explain the higher readmission rate in ESRD patients [33]. The higher readmissions and in-hospital complications could be due to a sicker patient population receiving LPM.

Several limitations should be acknowledged in our study. The retrospective nature of our analysis and reliance on administrative data introduce inherent biases and limitations associated with coding accuracy and data completeness. The NIS database lacks long-term follow-up data beyond readmission-related in-hospital data, precluding our ability to assess outcomes beyond hospitalizations. Although we used a multivariate regression model to adjust for differences in baseline characteristics between the groups, the LPM group had a higher prevalence of heart failure, atrial fibrillation, and coagulopathy compared to the TVPM group. This could explain the higher odds of complications observed in this group. Furthermore, compared to the TVPM population, the LPM population was more represented in large, teaching hospitals. Thus, important differences in healthcare workforce, including operator experience, might exist between the two populations. Despite these limitations, the study’s strength lies in the generalizability of our study compared to single-center studies, and the use of a reliable and extensively used database. The complex comorbidities in the patient population make a subset of the population not an eligible candidate for TVPM, which tends to enroll those complex patients with a higher risk of mortality and readmission in the LPM group. Despite these limitations, LPM seems safe and feasible with comparable in-hospital mortality and 30-day readmission with some higher odds of cardiovascular complications.

## 5. Conclusions

In conclusion, we used nationally representative data to compare real-world outcomes between TVPM and LPM implantation in ESRD patients, including in-hospital mortality, 30-day readmission rate, in-hospital complications, and healthcare resource utilization. The two cohorts had no significant difference regarding in-hospital mortality and 30-day rate of readmission. However, LPM admissions were associated with higher odds of cardiovascular complications and blood transfusion rates. Although our study on ESRD patients noted higher odds of short-term in-hospital complications and healthcare resource utilization, prior studies on non-ESRD populations have established significantly lower long-term complication rates with LPM than TVPM. Further research is necessary to gain a clearer understanding of these findings and to investigate potential strategies for reducing complications and healthcare resource utilization in ESRD patients undergoing LPM implantation, with the aim of improving outcomes and reducing the cost of care.

## Figures and Tables

**Table 1:** Baseline characteristics of ESRD patients who underwent pacemaker implantation.

Baseline Characteristics, *n* (%)	Total	TVPM	LPM	*p* Value
	*n* = 7865	*n* = 6384 (81.2%)	*n* = 1481 (18.8%)	
Female	3116 (39.6%)	2566 (40.2%)	550 (37.1%)	0.11
Age, years, median (IQR)	72 (65–80)	72 (65–80)	72 (64–79)	0.35
Median household income for patient’s ZIP				0.26
0–25th percentile	2600 (33.5%)	2130 (33.8%)	470 (32.2%)	
26th to 50th percentile (median)	2134 (27.5%)	1758 (27.9%)	376 (25.7%)	
51st to 75th percentile	1728 (22.3%)	1369 (21.7%)	359 (24.5%)	
76th to 100th percentile	1299 (16.7%)	1041 (16.5%)	258 (17.6%)	
Insurance				<0.01
Medicare	6822 (88.7%)	5553 (88.9%)	1269 (88.1%)	
Medicaid	340 (4.4%)	249 (4.0%)	92 (6.4%)	
Private insurance	500 (6.5%)	431 (6.9%)	768 (4.7%)	
Self-pay	28 (0.4%)	17 (0.3%)	11 (0.8%)	
Hospital Bed size				<0.01
Small	944 (12.0%)	846 (13.3%)	98 (6.6%)	
Medium	2074 (26.4%)	1743 (27.3%)	331 (22.4%)	
Large	4847 (61.6%)	3794 (59.4%)	1052 (71.0%)	
Teaching hospital	6133 (78.0%)	4809 (75.3%)	1324 (89.4%)	<0.01
Elective	468 (6.0%)	373 (5.9%)	95 (6.4%)	0.57
Comorbidities				
Hypertension	7537 (95.8%)	6124 (95.9%)	1413 (95.4%)	0.49
Diabetes mellitus	5640 (71.7%)	4579 (71.7%)	1061 (71.6%)	0.95
Peripheral vascular disease	934 (11.9%)	784 (12.3%)	150 (10.1%)	0.10
Coronary artery disease	3875 (49.3%)	3128 (49.0%)	747 (54.3%)	0.50
Heart failure	4162 (52.9%)	3295 (51.6%)	867 (58.5%)	<0.01
Lung disease	1849 (23.5%)	1480 (23.2%)	369 (24.9%)	0.32
Liver disease	491 (6.2%)	374 (5.9%)	117 (7.9%)	0.06
Obesity	1746 (22.2%)	1429 (22.4%)	317 (21.4%)	0.56
Smoker	2053 (26.1%)	1663 (26.1%)	390 (26.3%)	0.88
Malignancy	250 (3.2%)	201 (3.2%)	49 (3.3%)	0.85
Coagulopathy	1001 (12.7%)	780 (12.2%)	221 (14.9%)	0.05
Atrial fibrillation	1994 (25.4%)	1574 (24.7%)	420 (28.3%)	0.03

Abbreviations: TVPM, transvenous pacemaker; LPM, leadless pacemaker; IQR, interquartile range.

**Table 2:** In-hospital outcomes for index admissions for pacemaker implantation in ESRD patients.

Outcome, *n* (%)	Total	TVPM	LPM	aOR (95% CI)	*p* Value
Mortality	232 (3.0%)	178 (2.8%)	55 (3.7%)	1.32 (0.77–2.24)	0.31
Device complications *	344 (4.4%)	274 (4.3%)	70 (4.7%)	1.12 (0.72–1.74)	0.62
Cardiac complications +	59 (0.7%)	31 (0.5%)	27 (1.9%)	4.11 (1.69–10.03)	<0.01
Vascular complications ++	54 (0.7%)	32 (0.5%)	22 (1.5%)	3.57 (1.40–9.11)	<0.01
Blood transfusion	451 (5.7%)	324 (5.1%)	128 (8.6%)	1.85 (1.32–2.60)	<0.01
Respiratory complications §	494 (6.3%)	373 (5.8%)	121 (8.2%)	1.30 (0.92–1.84)	0.14
Acute kidney injury	903 (11.5%)	744 (11.7%)	158 (10.7%)	0.85 (0.64–1.13)	0.27
Length of stay, days, median (IQR)	4 (3–8)	4 (2–8)	5 (3–9)	-	<0.01
Total charges, $, median (IQR)	101,630 (67,060–158,779)	93,919 (62,778–146,722)	139,826 (96,775–213,083)	-	<0.01

* Thrombosis of the device, device infection, revision procedure. + Pericardial (hemopericardium, cardiac tamponade), myocardial (myocardial injury, cardiac perforation). ++ Vascular injury, fistula, hematoma. § Respiratory failure, mechanical ventilation, pneumothorax. Abbreviations: TVPM, transvenous pacemaker; LPM, leadless pacemaker; aOR, adjusted odds ratio; IQR, interquartile range; 95% CI, 95% confidence interval.

## Data Availability

All data related to the study are presented in the study and Supplementary Files.

## Supplementary Materials

The following supporting information can be downloaded at: https://www.mdpi.com/article/10.3390/jcm14010202/s1, Table S1: ICD-10 codes used to define variables in the study.
