# Supplementary material for: Leadless Pacemaker vs. Transvenous Pacemaker in End Stage Kidney Disease: Insights from the Nationwide Readmission Database
Source: J Clin Med. 2025 Jan 2;14(1):202. doi: 10.3390/jcm14010202 (PMC11721035; doi:10.3390/jcm14010202)
Supplement: Supplementary file 1 [file jcm-14-00202-s001.zip › jcm-3387948-supplementary.pdf]

Supplementary Table S1: ICD-10 codes used to define variables in the study

Variable	ICD-10 code	
ESRD	CM	N186, Z992, Z9115
Sick sinus syndrome	CM	I495
Atrial fibrillation, atrial flutter		I480, I481, I4811, I4819, I482, I4820, I4821, I4891, I483, I484, I4892
Bradycardia	CM	R001
Conduction disorder	CS	I440, I441, I442, I4430, I4439, I444, I445, I4460, I4469, I447, I450, I4510, I4519, I452, I453, I454, I455, I4589, I459
h/o CIED	CM	Z950, Z95810
Coronary artery bypass grafting	PCS	0210093 0210098 0210099 021009C 021009F 021009W 02100A3 02100A8 02100A9 02100AC 02100AF 02100AW 0211093 0211098 0211099 021109C 021109F 021109W 02110A3 02110A8 02110A9 02110AC 02110AF 02110AW 0212093 0212098 0212099 021209C 021209F 021209W 02120A3 02120A8 02120A9 02120AC 02120AF 02120AW 0213093 0213098 0213099 021309C 021309F 021309W 02130A3 02130A8 02130A9 02130AC 02130AF 02130AW
Catheter ablation	PCS	02563ZZ 02573ZZ 02583ZZ 025T3ZZ 025S3ZZ
Valve procedures	PCS	024F0xJ (7, 8, J, K), 024G0xJ (7, 8, J, K), 024J0xJ (7, 8, J, K), 025FxZZ (0, 3, 4), 025GxZZ (0, 3, 4), 025HxZZ (0, 3, 4), 025JxZZ (0, 3, 4), 027F0xZ (4, D, Z), 027F3xZ (4, D, Z), 027F4xZ (4, D, Z), 027G0xZ (4, D, Z), 027G3xZ (4, D, Z), 027G4xZ (4, D, Z), 027H0xZ (4, D, Z), 027H3xZ (4, D, Z), 027H4xZ (4, D, Z), 027J0xZ (4, D, Z), 027J3xZ (4, D, Z), 027J4xZ (4, D, Z), 02BF0Zx (X, Z), 02BF3Zx (X, Z), 02BF4Zx (X, Z), 02BG0Zx (X, Z), 02BG3Zx (X, Z), 02BG4Zx (X, Z), 02BH0Zx (X, Z), 02BH3Zx (X, Z), 02BH4Zx (X, Z), 02BJ0Zx (X, Z), 02BJ3Zx (X, Z), 02BJ4Zx (X, Z), 02CFxZZ (0, 3, 4), 02CGxZZ (0, 3, 4), 02CHxZZ (0, 3, 4), 02CJxZZ (0, 3, 4), 02NFxZZ (0, 3, 4), 02NGxZZ (0, 3, 4), 02NHxZZ (0, 3, 4), 02NJxZZ (0, 3, 4), 02QF0Zx (J, Z), 02QF3Zx (J, Z), 02QF4Zx (J, Z), 02QG0Zx (J, Z), 02QG3Zx (J, Z), 02QG4Zx (J, Z), 02QH0Zx (J, Z), 02QH3Zx (J, Z), 02QH4Zx (J, Z), 02QJ0Zx (J, Z), 02QJ3Zx (J, Z), 02QJ4Zx (J, Z), 02RF0xZ

		(7, 8, J, K), 02RF3xx (7H, 7Z, 8H, 8Z, JH, JZ, KH, KZ), 02RF4xZ (7, 8, J, K), 02RG0xZ (7, 8, J, K), 02RG3xx (7H, 7Z, 8H, 8Z, JH, JZ, KH, KZ), 02RG4xZ (7, 8, J, K), 02RH0xZ (7, 8, J, K), 02RH3xx (7H, 7Z, 8H, 8Z, JH, JZ, KH, KZ), 02RH4xZ (7, 8, J, K), 02RJ0xZ (7, 8, J, K), 02RJ3xx (7H, 7Z, 8H, 8Z, JH, JZ, KH, KZ), 02RJ4xZ (7, 8, J, K), 02UF0xx (7J, 7Z, 8J, 8Z, JJ, JZ, KJ, KZ), 02UF3xx (7J, 7Z, 8J, 8Z, JJ, JZ, KJ, KZ), 02UF4xx (7J, 7Z, 8J, 8Z, JJ, JZ, KJ, KZ), 02UG0xx (7J, 7Z, 8J, 8Z, JJ, JZ, KJ, KZ), 02UG3xx (7J, 7Z, 8J, 8Z, JJ, JZ, KJ, KZ), 02UG4xx (7J, 7Z, 8J, 8Z, JJ, JZ, KJ, KZ), 02UH0xx (7J, 7Z, 8J, 8Z, JJ, JZ, KJ, KZ), 02UH3xx (7J, 7Z, 8J, 8Z, JJ, JZ, KJ, KZ), 02UH4xx (7J, 7Z, 8J, 8Z, JJ, JZ, KJ, KZ), 02UJ0xx (7J, 7Z, 8J, 8Z, JJ, JZ, KJ, KZ), 02UJ3xx (7J, 7Z, 8J, 8Z, JJ, JZ, KJ, KZ), 02UJ4xx (7J, 7Z, 8J, 8Z, JJ, JZ, KJ, KZ)
Percutaneous coronary intervention	PCS	0203xx, 02704xx, 02713xx, 02714xx, 02723xx, 02724xx, 02733xx, 02734xx, 02C03xx, 02C04xx, 02C13xx, 02C14xx, 02C23xx, 02C24xx, 02C33xx, 02C34xx
Leadless pacemaker	PCS	02HK3NZ
Transvenous pacemaker	PCS	0JH604Z 0JH634Z 0JH804Z 0JH834Z 0JH605Z 0JH635Z 0JH805Z 0JH835Z 0JH606Z 0JH636Z 0JH806Z 0JH836Z 02HK4JZ 02HK3JZ 02H64JZ 02H63JZ
Device thrombus	CM	T82867A, T82868A
Device infection	CM	T827xxA
Mech comp of CED-breakdown, displacement	CM	T821xxA
Device revision	PCS	02WA0MZ, 02WA3MZ, 02WA4MZ, 02WAXMZ 0JWT0PZ, 0JWT3PZ, 0JWTXPZ 02WA0NZ, 02WA3NZ, 02WA4NZ, 02WAXNZ
Cardiac complications	CM	I314 I312 S260xxA S261xxA S269xxA I9751)
	PCS	0W9D00Z, 0W9D0ZZ, 0W9C00Z, 0W9C0ZZ 0W9D30Z, 0W9D3ZZ, 0W9D40Z, 0W9D4ZZ, 0W9C30Z, 0W9C3ZZ, 0W9C40Z, 0W9C4ZZ 0W3D0ZZ, 0W3D3ZZ, 0W3D4ZZ

Vascular complications	CM	S15* S25* S35* S45* S55* S65* S75* S85* I772 T8183XA<br>I770 I97621 I97630 I97631 I97638
Blood transfusion	PCS	30230H0, 30230H1, 30230N0, 30230N1, 30230P0, 30230P1, 30233H0, 30233H1, 30233N0, 30233N1, 30233P0, 30233P1, 30240H0, 30240H1, 30240N0, 30240N1, 30240P0, 30240P1, 30243H0, 30243H1, 30243N0, 30243N1, 30243P0, 30243P1, 30250H0, 30250H1, 30250N0, 30250N1, 30250P0, 30250P1, 30253H0, 30253H1, 30253N0, 30253N1, 30253P0, 30253P1, 30260H0, 30260H1, 30260N0, 30260N1, 30260P0, 30260P1, 30263H0, 30263H1, 30263N0, 30263N1, 30263P0, 30263P1, 30273H1, 30273N1, 30273P1, 30277H1, 30277N1, 30277P1
Respiratory complications	CM	J95.821 J95.81
	PCS	5A1955Z 5A1935Z 5A1945Z
AKI	CM	N170, N171, N172, N178, N179
Hypertension	CM	Elixhauser comorbidity mapping
Diabetes Mellitus	CM	Elixhauser comorbidity mapping
Peripheral Vascular Disease	CM	Elixhauser comorbidity mapping
Coronary Artery Disease	CM	I251*, I252, I255, I256, I257*, I258*, I259
Heart Failure	CM	Elixhauser comorbidity mapping
Lung Disease	CM	Elixhauser comorbidity mapping
Liver Disease	CM	Elixhauser comorbidity mapping
Obesity	CM	Elixhauser comorbidity mapping
Smoker	CM	Z87891 F17200 F17203 F17201 F17208 F17209
Malignancy	CM	Elixhauser comorbidity mapping
Coagulopathy	CM	Elixhauser comorbidity mapping
